# Causal relationship between type 2 diabetes and glioblastoma: bidirectional Mendelian randomization analysis

**DOI:** 10.1038/s41598-024-67341-x

**Published:** 2024-07-17

**Authors:** Wei Chen, Taoyuan Zhang, Hui Zhang

**Affiliations:** 1 Department of Neurosurgery, Xi’an People’s Hospital (Xi’an Fourth Hospital), Xi’an, 710100 Shaanxi China; 2 Department of Anesthesiology and Perioperative Medicine, Xijing Hospital, the Fourth Military Medical University, Xi’an, 710032 Shaanxi China

**Keywords:** Glioblastoma, Type 2 diabetes mellitus, Mendelian randomization, Genome-Wide Association Studies, CNS cancer, Cancer epigenetics, Risk factors

## Abstract

As the prevalence of Type 2 Diabetes Mellitus (T2DM) and Glioblastoma (GBM) rises globally, the relationship between T2DM and GBM remains controversial. This study aims to investigate whether genetically predicted T2DM is causally associated with GBM. We performed bidirectional Mendelian randomization (MR) analysis using data from genome-wide studies on T2DM (N = 62,892) and GBM (N = 218,792) in European populations. The results of the inverse-variance weighted (IVW) approach served as the primary outcomes. We applied Cochran’s Q test and MR-Egger regression for heterogeneity assessment. Leave-one-out analysis was used to evaluate whether any single SNP significantly influenced the observed effect. Our findings reveal a significant causal association between T2DM and an increased risk of GBM (OR [95% CI] 1.70 [1.09, 2.65], P = 0.019). Conversely, the reverse association between T2DM and GBM was insignificant (OR [95% CI] 1.00 [0.99, 1.01], P = 0.408) (*P* > 0.40). Furthermore, the results from Cochran’s Q-test and funnel plots in the MR-Egger method indicated no evidence of pleiotropy between the SNPs and GBM. Additionally, we mapped causal SNPs to genes and identified 10 genes, including *MACF1, C1orf185, PTGFRN, NOTCH2, ABCB10, GCKR, THADA, RBMS1, SPHKAP*, and *PPARG*, located on chromosomes 1, 2, and 3. These genes are involved in key biological processes such as the BMP signaling pathway and various metabolic pathways relevant to both conditions. This study provides robust evidence of a significant causal relationship between T2DM and an increased risk of GBM. The identified SNP-mapped genes highlight potential biological mechanisms underlying this association.

## Introduction

Gliomas are a fatal type of primary brain tumor that can occur anywhere in the central nervous system (CNS)^1^. Glioblastoma (GBM) accounts for over 40% of malignant glioma cases^2^. The world health organization (WHO) classification system groups gliomas into 4 grades (localized grade 1; diffuse grades 2–4), defined by increasing degrees of undifferentiation, anaplasia, and aggressiveness^3^. GBM is referred to as grade 4 and is highly aggressive^4^. The incidence of GBM is 7 per 100,000 population and is partly influenced by factors such as lifestyle, obesity, and diabetes mellitus^5–7^. Diabetes mellitus, a metabolic condition, is associated with an increased risk of various cancers, including kidney, pancreatic, cervical, and uterine cancer^8^.

Type 2 diabetes mellitus (T2DM) is a global health burden and represents more than 90% of patients with diabetes^9^. Despite studies showing a positive association between T2DM and the development of various cancer types^10–12^, the association between T2DM and GBM remains controversial due to possible residual confounding and reverse causality in observational studies^13^. One meta-analysis indicates that DM is associated with a decreased risk of gliomas. Nonetheless, this study lacked a clear distinction between brain cancer, glioma, and type 1 DM or type 2 DM^14^. Conversely, one study indicates that diabetes mellitus (DM) might potentially result in a two-fold increase in the incidence of GBM in white populations compared to black individuals^13^. Furthermore, several studies found no significant associations between T2DM and the risk of glioma^15,16^. As the prevalence of GBM and T2DM is rapidly on the rise, it is vital to elucidate the causal relationship between them for the treatment and prevention of these diseases.

Mendelian randomization (MR) is a genetic method for inferring the causal effect of an exposure on an outcome^17^. The MR design minimizes confounding from environmental factors as alleles are randomly assigned during conception. Furthermore, it mitigates bias from reverse causation since the disease cannot impact the genotype^18,19^. The increasing prevalence of T2DM and GBM poses significant public health challenges. While T2DM is known to be associated with various cancers, the causal relationship between T2DM and GBM remains controversial^11,20^. Previous observational studies have been limited by residual confounding and reverse causality^13,21^. This study is novel in its bidirectional MR to explore the causal relationship between T2DM and GBM. By using the most extensive GWAS datasets available, this study provides new insights and robust evidence that may have important implications for the prevention and treatment of these conditions.

## Materials and methods

### Study design

**Figure 1:**
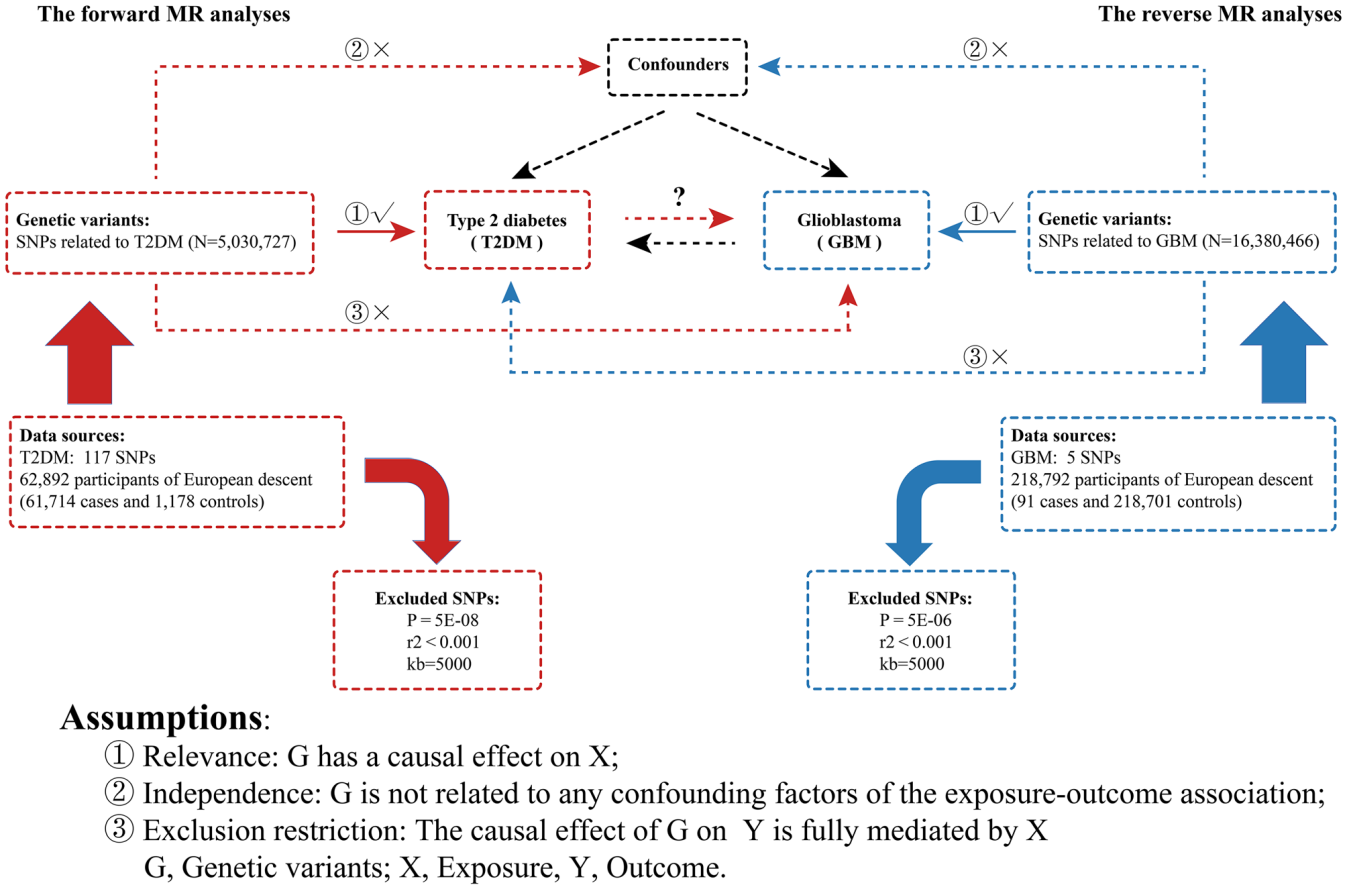
The three core MR assumptions and study design. The forward MR was marked in red, with T2DM as exposure and GBM as the outcome. The reverse MR was marked in blue, with GBM as exposure and T2DM as the outcome.

The core Mendelian Randomization (MR) assumptions and study design are depicted in Fig. 1. In the forward MR analyses, T2DM was treated as the exposure and GBM as the outcome, while in the reverse MR analyses, GBM was considered the exposure and T2DM the outcome. All datasets used in this study were obtained from genome-wide association studies (GWAS) databases, and no ethical approval was required.

### Instrumental variable selection for MR

#### Forward MR analysis (T2DM as exposure)

The glioblastoma cases in this study were classified according to the WHO classification 2016. We acknowledge that the 2021 WHO classification includes Astrocytoma IDH-mutant grade 4 under the glioblastoma category, but our analysis did not differentiate these subtypes. For the forward MR analysis, we used genome-wide significant SNPs with a p-value threshold of 5e-8 to ensure the robustness of the instrumental variables. Genetic variants associated with T2DM (GWAS ID: ebi-a-GCST006867) were obtained from the GWAS summary data, which included 61,714 cases and 117,8 controls with 5,030,727 SNPs.

#### Reverse MR analysis (GBM as exposure)

For the reverse MR analysis, due to the limited number of SNPs available, we used a more lenient threshold of 5e-6. This approach was taken to ensure enough instrumental variables while maintaining the robustness of our findings. Genetic variants associated with GBM (GWAS ID: finn-b-C3_GBM) were derived from GWAS summary data, consisting of 91 cases and 218,701 controls with 16,380,466 SNPs.

### Data harmonization

To ensure compatibility and consistency, we harmonized the allelic directions of filtered SNPs in exposure and outcome datasets. Incompatible and palindromic SNPs were removed, ensuring that the effect allele (along with the corresponding beta and effect allele frequency) within the outcome dataset reflected the same effect allele as in the exposure data. Additionally, we manually reviewed the remaining SNPs to eliminate any associated with the outcomes and exposures. We selected 123 SNPs associated with T2DM as instrumental variables. After harmonization analysis, 6 SNPs were excluded, resulting in 117 SNPs being included in the MR analyses (Fig. 1 and Supporting Information: Table 1). Nine SNPs associated with GBM were initially selected as instrumental variables (Fig. 1 and Supporting Information: Table 2).

### Two-sample MR analysis

MR was implemented in R (version 4.1.3) using the “TwoSampleMR” package. The random-effects model of IVW was considered the primary method^22^. For robust causal inference, causal associations required both IVW P < 0.05^23^. In MR studies, accuracy is assessed through statistical measures such as odds ratios (ORs) with confidence intervals (CIs) to indicate the strength and significance of the causal association. We also conducted sensitivity analyses, including Cochran’s Q test and MR-Egger regression, to assess heterogeneity and pleiotropy, ensuring the robustness and validity of our findings^24^. The leave-one-out analysis was performed to ensure that the observed effect was not significantly influenced by any single SNP.

### SNP-to-gene mapping and enrichment analysis

To further investigate the potential biological mechanisms underlying our findings, we utilized the “biomaRt” package in R software. Specifically, we integrated all harmonized T2DM-associated SNPs and employed the MAGMA (Multi-marker Analysis of Genomic Annotation) tool to map these SNPs to their corresponding genes. Furthermore, we conducted Gene Ontology (GO) and Kyoto Encyclopedia of Genes and Genomes (KEGG) enrichment analyses on the GWAS genotype data using the “clusterProfiler” package in R software.

## Results

### The causal effect of T2DM on GBM

**Figure 2:**
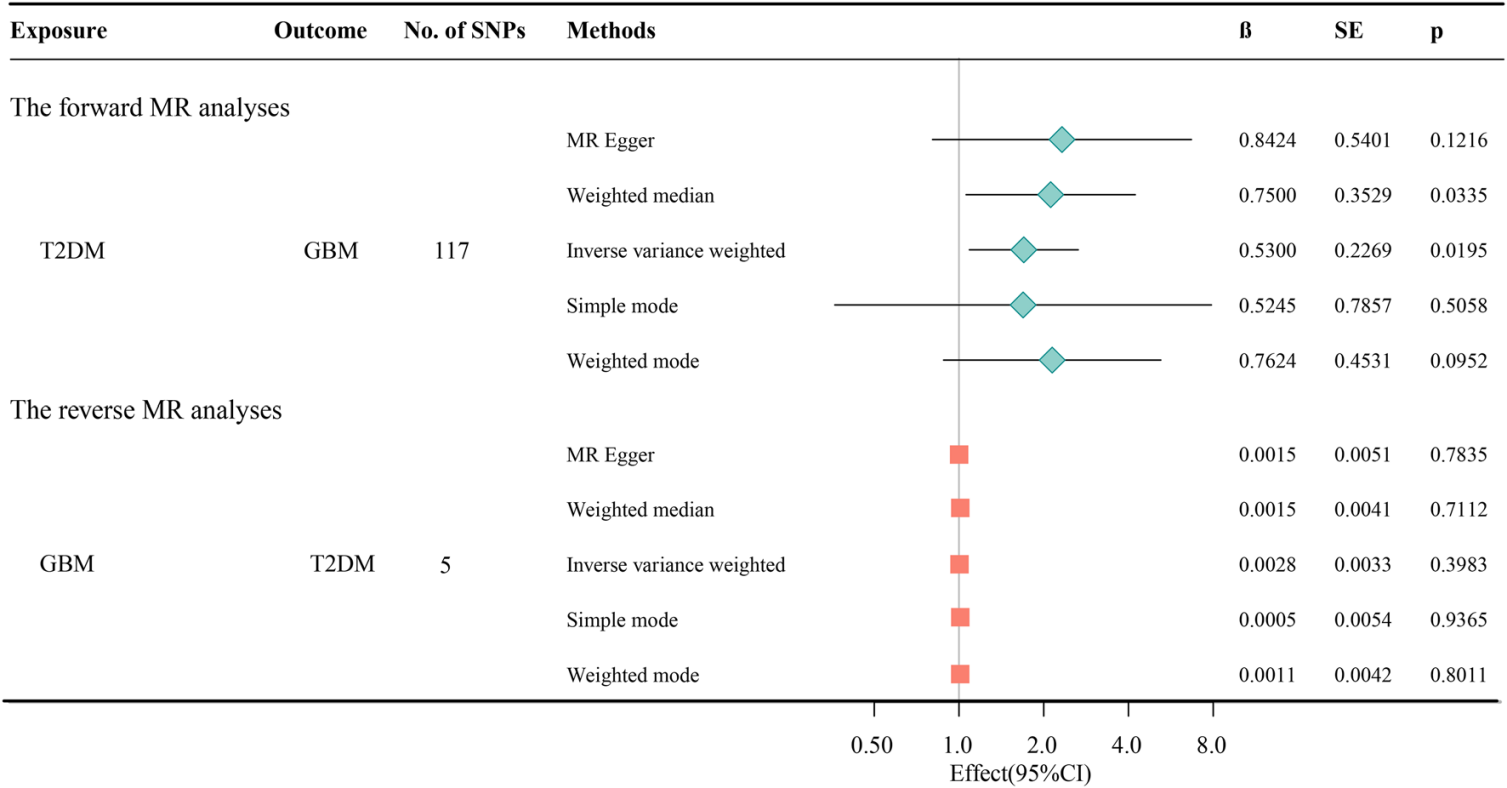
T2DM and its association with GBM in the MR analyses.

**Figure 3:**
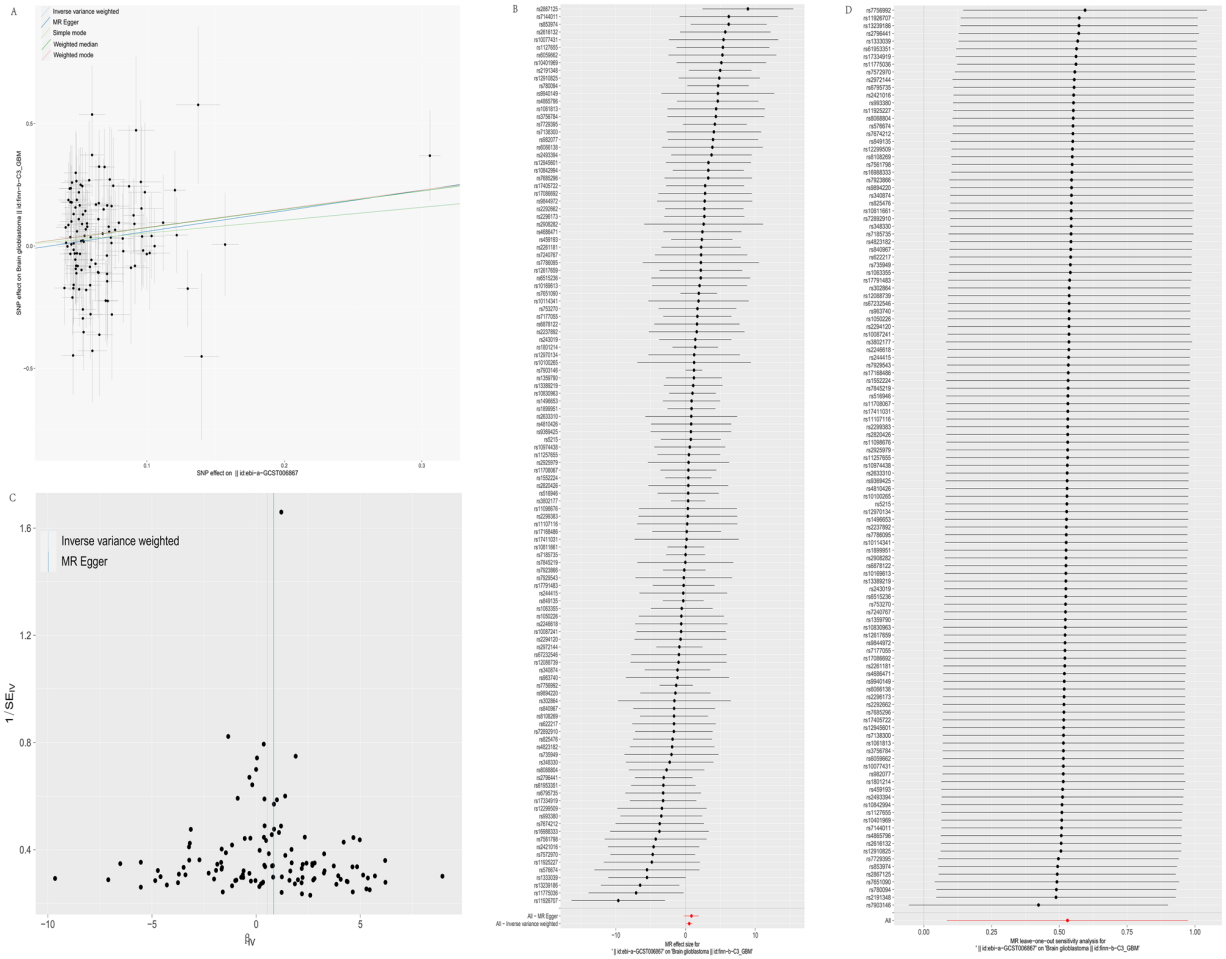
The forward MR analyses: (**A**) Scatter plot between T2DM and GBM; (**B**) A forest plot for each SNP; (**C**) Funnel plot; (**D**) Leave-one-out analyses.

In the forward MR analyses, the IVW method indicated a significant causal association between T2DM and an increased risk of GBM (OR [95% CI] 1.70 [1.09, 2.65], *P* = 0.019) (Fig. 2 and Fig. 3A,B). The other four methods, including MR-Egger regression, Weighted mode, Simple mode, and Weighted median, showed no significant association between T2DM and the risk of GBM (*P* > 0.05). The results of Cochran’s Q-test in MR-Egger (Q = 120.142, *P* = 0.353) or IVW (Q = 120.566, *P* = 0.3673) and funnel plots revealed no pleiotropy between SNPs and GBM (*P* > 0.05) (Table 1, Fig. 3C). However, the leave-one-out analysis confirmed that the causal association was not influenced by any single genetic variant, supporting the robustness of the IVW results (Fig. 3D). In total, all the results indicated a positive association between T2DM and the risk of GBM.

**Table 1:** Heterogeneity and Pleiotropy analyses.

Exposure	Outcome	No. of SNPs	MR‐Egger regression		Heterogeneity analyses		
			Intercept	p_intercept	Method	Q	Q_pval
The forward MR analyses							
T2DM	GBM	117	− 0.0245	0.5249	MR Egger	120.1420	0.3527
					Inverse variance weighted	120.5669	0.3671
The reverse MR analyses							
GBM	T2DM	5	0.0023	0.7610	MR Egger	2.2123	0.5295
					Inverse variance weighted	2.3232	0.6765

### SNP-to-gene mapping and enrichment analysis

**Figure 4:**
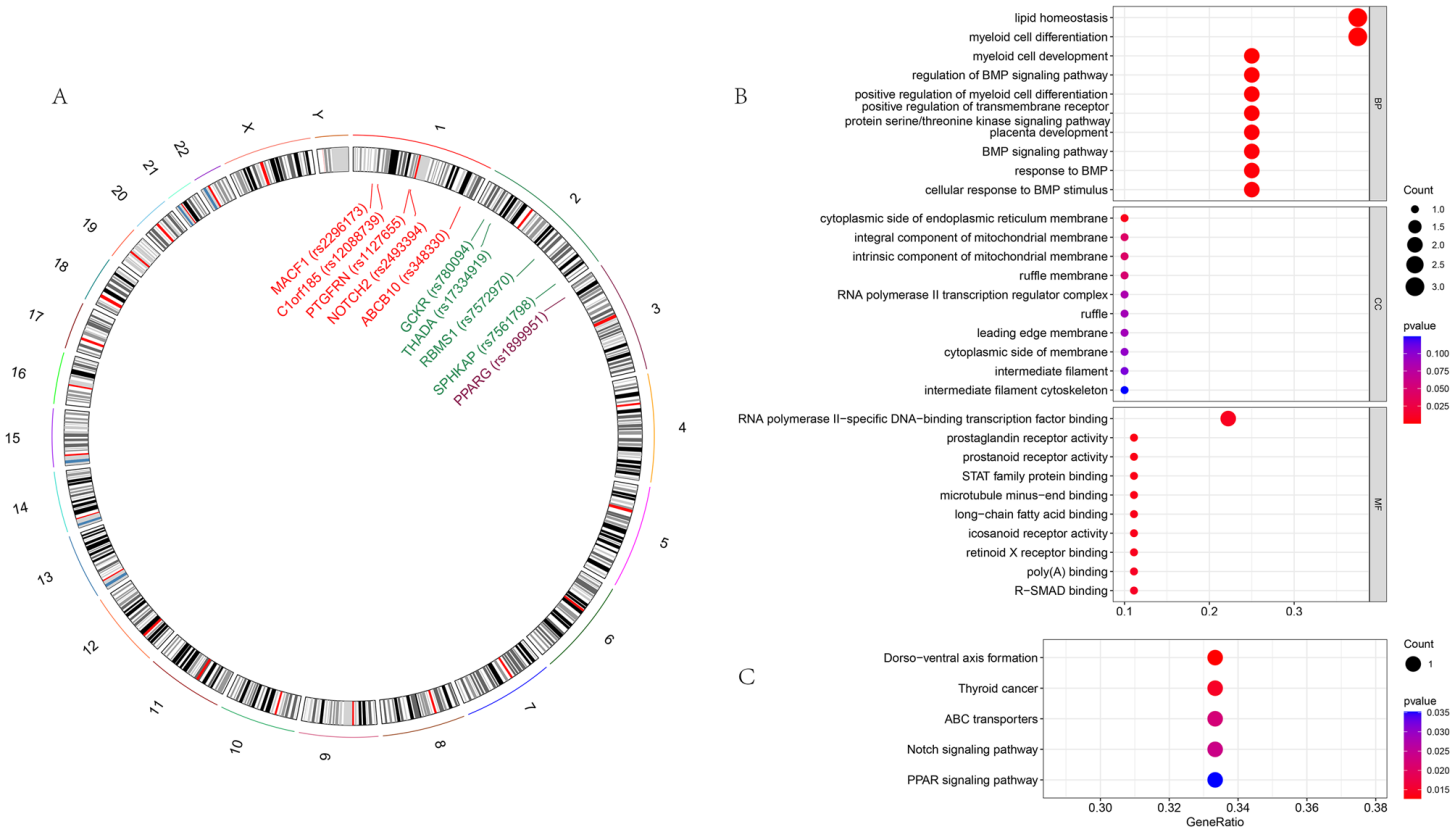
SNP-mapped genes and molecular pathways. (**A**) SNP-to-gene mapping revealed the genes corresponding to the forward SNPs. The SNPs and their corresponding genes are labeled in the figure. (**B**, **C**) Enrichment analysis revealed the molecular functions of the mapped genes (GO, KEGG).

To identify the genes potentially influenced by SNPs associated with both T2DM and GBM, we mapped the causal SNPs to genes. In total, 10 genes were identified from 117 harmonized SNPs, located on chromosomes 1, 2, and 3, including key genes such as *MACF1, C1orf185, PTGFRN, NOTCH2, ABCB10, GCKR, THADA, RBMS1, SPHKAP*, and *PPARG* (Fig. 4A). To further explore the molecular function of these genes, we performed gene functional enrichment. The results reveal that the genes are significantly enriched in several biological processes, including cellular response to BMP stimulus, BMP signaling pathway, response to BMP, placenta development, positive regulation of transmembrane receptor protein serine/threonine kinase signaling pathway, and various myeloid cell processes (Fig. 4B). The results of KEGG pathway enrichment analysis indicate significant enrichment of genes in the PPAR signaling pathway, Notch signaling pathway, ABC transporters, thyroid cancer pathway, and dorso-ventral axis formation pathway (Fig. 4C).

### The causal effect of GBM on T2DM

**Figure 5:**
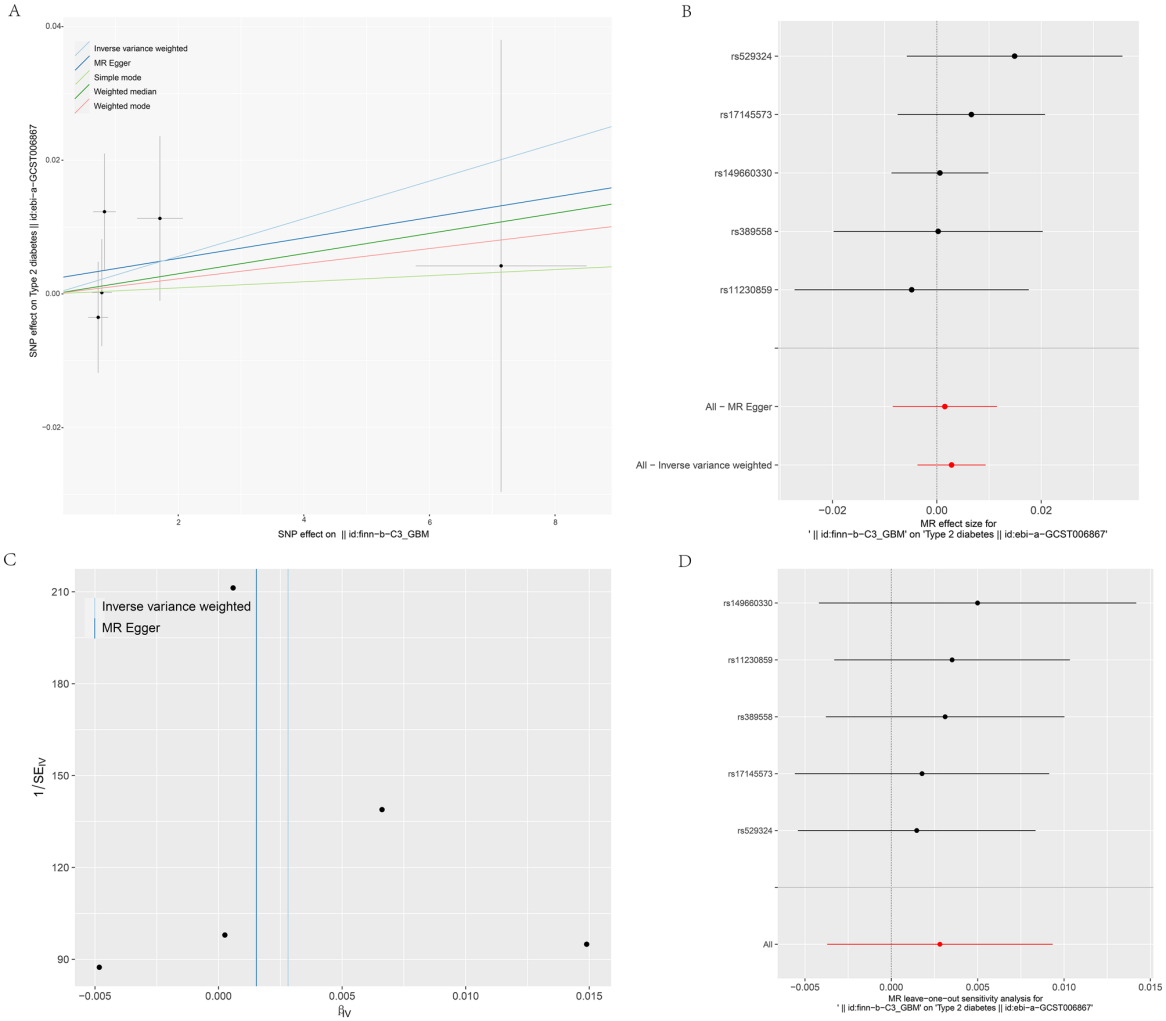
The reverse MR analyses: (**A**) Scatter plot between GBM and T2DM; (**B**) A forest plot for each SNP; (**C**) Funnel plot; (**D**) Leave‐one‐out analyses.

In the reverse MR analyses, the results indicated no significant association between GBM and the risk of T2DM in five methods, including MR-Egger regression, Weighted mode, Simple mode, Weighted median, and IVW (OR [95% CI] of IVW: 1.00 [0.99, 1.01], *P* > 0.40) (Fig. 2, Fig. 5A, B). The Cochran’s Q (Q = 2.212, *P* = 0.529) test and MR-Egger (Q = 2.323, *P* = 0.677) regression did not reveal any evidence supporting horizontal pleiotropy and heterogeneity (*P* > 0.05) (Table 1, Fig. 5C). The leave-one-out analysis did not identify any SNP significantly influencing the overall estimate (Fig. 5D). Collectively, these results suggest that the reverse causal association between T2DM and GBM was insignificant.

## Discussion

In this study, we used bidirectional MR analyses to investigate the causal relationship between T2DM and GBM. Our primary analysis using the IVW method indicated a significant causal association between T2DM and an increased risk of GBM (OR [95% CI] 1.70 [1.09, 2.65], P = 0.019). While the IVW method indicated a significant association (P = 0.02), the sensitivity analyses did not show significant results, suggesting that the causal relationship between T2DM and GBM may not be very strong. This discrepancy can be attributed to the different assumptions and statistical power of these methods. This highlights the need for further research to explore this association in different populations and use larger datasets to confirm these findings. However, the leave-one-out analysis confirmed that the causal association was not influenced by any single genetic variant, supporting the robustness of the IVW results. Despite the lack of significance in some sensitivity analyses, the IVW method remains a powerful and reliable approach for detecting causal associations in MR studies. Our findings suggest that T2DM and GBM may share some common clinical and pathophysiological characteristics.

Previous studies have indicated that up to 16% of individuals diagnosed with GBM have T2DM, aligning with our findings that T2DM increases the risk of GBM^25,26^. These results are consistent with research linking T2DM to an increased cancer risk, though associations specifically with GBM have been less explored^5,27^. Our study contributes a comprehensive analysis of the correlation between T2DM and an elevated risk for GBM and constitutes a significant advancement over previous studies^28^. The result was consistent with one study that found an elevated risk of brain tumors in individuals with diabetes^29^. Additionally, our study contrasts with some studies, which have reported negative or null associations between T2DM and GBM or brain cancers^13,15,21,30–32^. A relatively recent study found that T2DM was associated with a decreased risk of GBM^33^. Other studies revealed that T2DM and non-T2DM had similar risks of GBM^34,35^. This discrepancy might be due to differences in population characteristics, study design, or cancer subtypes examined^20^.

SNPs located in a gene or a regulatory region near a gene can directly affect the gene’s function and contribute to disease^36^. We identified 10 genes across chromosomes 1, 2, and 3: *MACF1, C1orf185, PTGFRN, NOTCH2, ABCB10, GCKR, THADA, RBMS1, SPHKAP*, and *PPARG*. Genes such as *GCKR* and *PPARG* emphasize the metabolic connection between T2DM and GBM. GCKR, influencing glucose metabolism, has variations associated with diabetes risk, while PPARG plays a pivotal role in adipogenesis and insulin sensitivity, highlighting its importance in T2DM^37,38^. Functional enrichment analysis revealed that these genes are significantly involved in critical biological processes, such as cellular response to BMP stimulus, BMP signaling pathway, placenta development, and various myeloid cell processes. The BMP signaling pathway, essential for cellular differentiation and proliferation, is relevant to both T2DM and GBM pathophysiology^39^. NOTCH2, part of the Notch signaling pathway, regulates cell fate decisions and is linked to both T2DM and GBM development^40^. The PPAR signaling pathway, crucial in lipid metabolism and inflammation, is central to T2DM and connected to cancer progression^41^.

The potential mechanisms underlying the association between T2DM and GBM may involve the complex interplay between metabolic disruptions in T2DM and the development of GBM. Hyperglycemia, a hallmark of T2DM, exacerbates oxidative stress and DNA damage, potentially inducing cancer development^42^. Elevated levels of insulin and insulin-like growth factor-1 (IGF-1) associated with T2DM have mitogenic effects on cancer cells, promoting proliferation and inhibiting apoptosis ^43,44^. Chronic inflammation, another consequence of T2DM, is recognized as a vital factor in cancer development^12^. Inflammatory cytokines and chemokines can promote GBM cell migration, angiogenesis, and invasion^45^. The synergy between hyperglycemia, insulin resistance, and chronic inflammation in T2DM may play a critical role in understanding the increased risk of GBM.

In contrast to traditional observational research, the primary advantage of our study lies in the use of MR, which can avoid confounding bias and reverse causality. Our findings have important implications for the prevention and treatment of both T2DM and GBM. Given the rising prevalence of T2DM and GBM, understanding their causal relationship can inform targeted interventions to mitigate the risk of GBM among individuals with T2DM.

There were several limitations in our study. Firstly, our findings are based on European populations and may not be generalizable to other ethnic groups. Secondly, although MR minimizes confounding, it cannot completely rule out pleiotropy, where genetic variants affect the outcome through pathways other than exposure. Finally, the bidirectional MR approach relies on the availability and quality of GWAS data, which can impact the robustness of the results.

## Conclusion

Our study suggests that T2DM is causally associated with an increased risk of GBM. This finding provides valuable evidence for the potential impact of T2DM on GBM development, which could be significant for both treatment and prevention strategies. Further studies are necessary to explore the relevance of this association across different populations and to investigate the underlying biological mechanisms. This additional research could enhance our understanding of the complex relationship between T2DM and GBM, ultimately leading to more effective interventions and improved patient outcomes.

### Supplementary Information

Supplementary Table 1.

Supplementary Table 2.

## Data Availability

The datasets generated and analyzed during the current study are available in the GWAS summary data repository at https://gwas.mrcieu.ac.uk/.

## Abbreviations

T2DM: Type 2 diabetes mellitus
GBM: Glioblastoma
MR: Mendelian randomization
IVW: Inverse variance-weighted
OR: Odds ratio
CI: Confidence interval
SNP: Single nucleotide polymorphism
GWAS: Genome-wide association studies
WHO: World health organization
IGF-1: Insulin-like growth factor-1
CNS: Central nervous system
GO: Gene ontology
KEGG: Kyoto encyclopedia of genes and genomes
